# Supplementary material for: A T7 RNAP regulatory toolbox for cell-free network engineering and biosensing applications
Source: Nat Commun. 2026 May 28;17:6941. doi: 10.1038/s41467-026-73811-9 (PMC13389190; doi:10.1038/s41467-026-73811-9)
Supplement: Supplementary file 1 — Supplementary Information [file 41467_2026_73811_MOESM1_ESM.pdf]

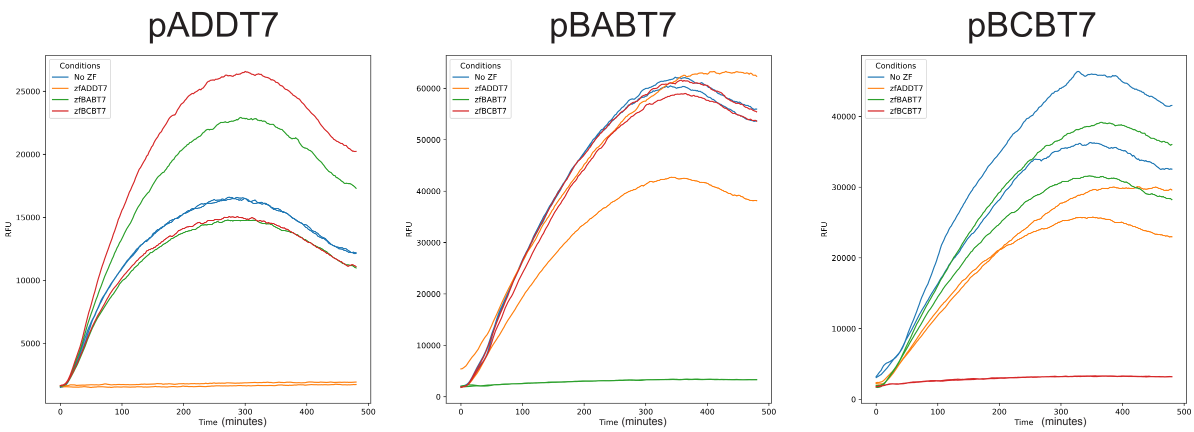

Supplementary Fig.1. Time-course of T7 promoter variant transcription under regulation of different repressors: Time-course of transcription from the three different promoters in the presence of different ZF_PR-ZF_T7 repressors showing specific and potent repression by the matched repressor (n=2), the results show strong and specific repression with minimal cross-reactivity. The curves were processed by moving average with the window size of 5. Source data are provided as a Source Data file.

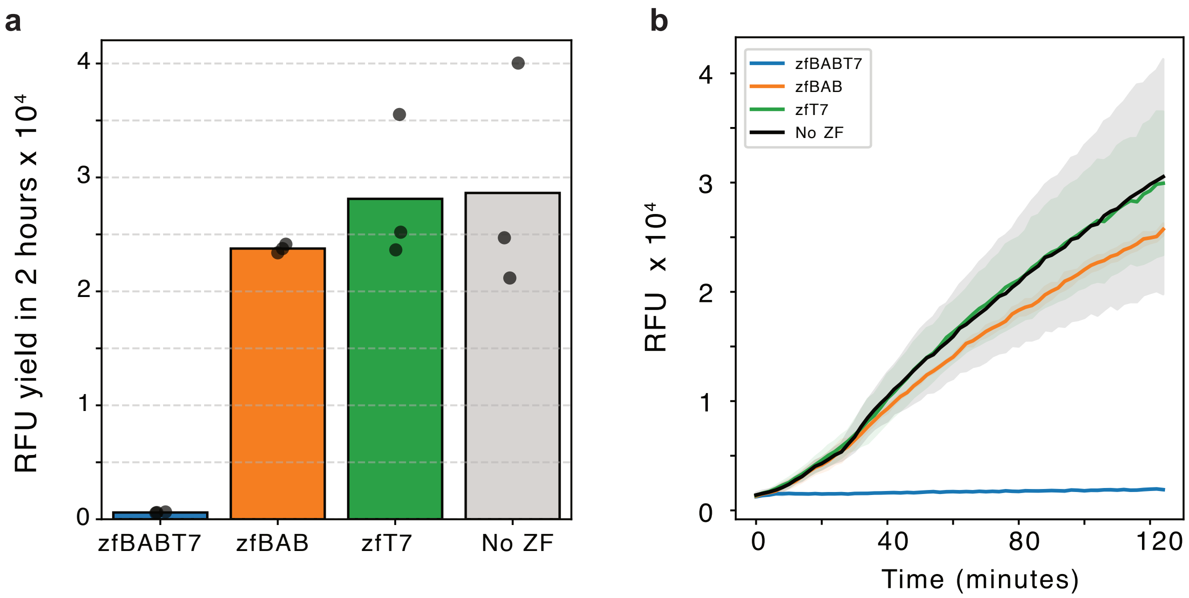

Supplementary Fig.2. Comparison between zfBABT7, zfBAB and zfT7: The zfBABT7, zfBAB, zfT7 are pre-expressed and added to PURExpress with pzfBABT7-pepper reporter. **a,** Comparison of repression efficiency across ZF variants. The single-domain ZFs zfBAB and zfT7 did not exhibit significant repression, whereas zfBABT7 strongly repress gene expression. (n=3) **b,** Time-course fluorescent profile of different ZFs.(n = 3; shaded regions indicate standard deviation). Source data are provided as a Source Data file.

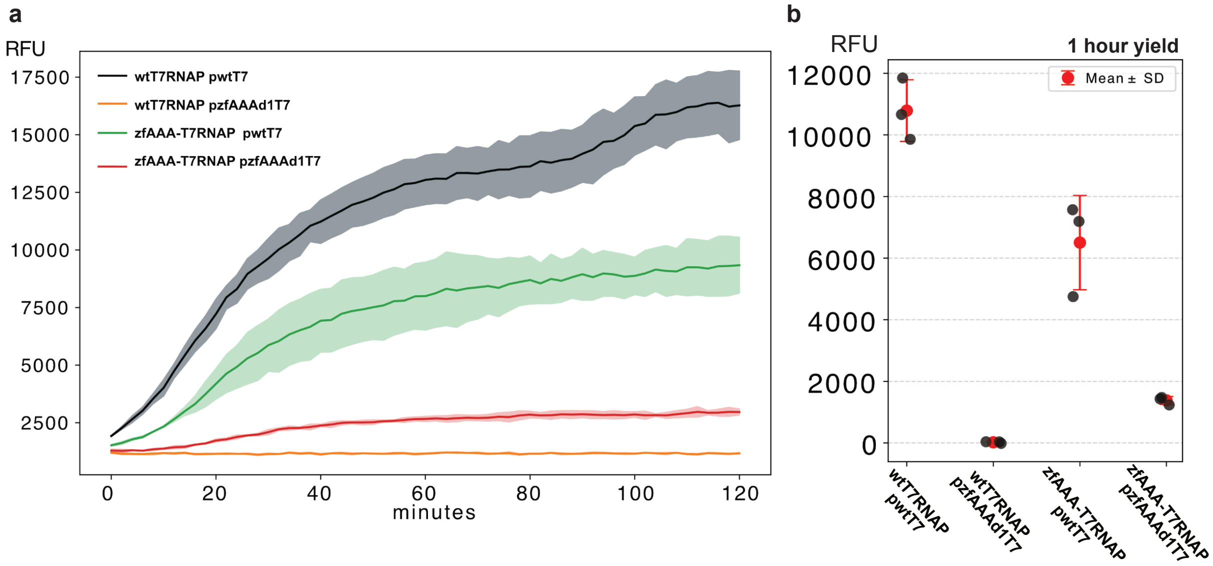

Supplementary Fig.3. Comparison of wild-type T7 RNAP and zfAAA–T7 RNAP on the wild-type T7 promoter and the engineered pzfAAAd1T7 promoter: IVT reactions were performed with 20 nM of either wild-type T7 RNAP or zfAAA–T7 RNAP and 0.5 nM Broccoli DNA templates with either the wild-type promoter or the pzfAAAd1T7 promoter. **a,** Time-course fluorescence traces under the four tested conditions. (n = 3; shaded regions indicate standard deviation) **b,** Broccoli RNA yield after 1 h. No detectable transcriptional leakiness of wild-type T7 RNAP was observed on the pzfAAAd1T7 promoter. Transcription by zfAAA–T7 RNAP on the pzfAAAd1T7 promoter is approximately 6–7-fold weaker than wild-type T7 RNAP on the wild-type promoter (n=3). Source data are provided as a Source Data file.

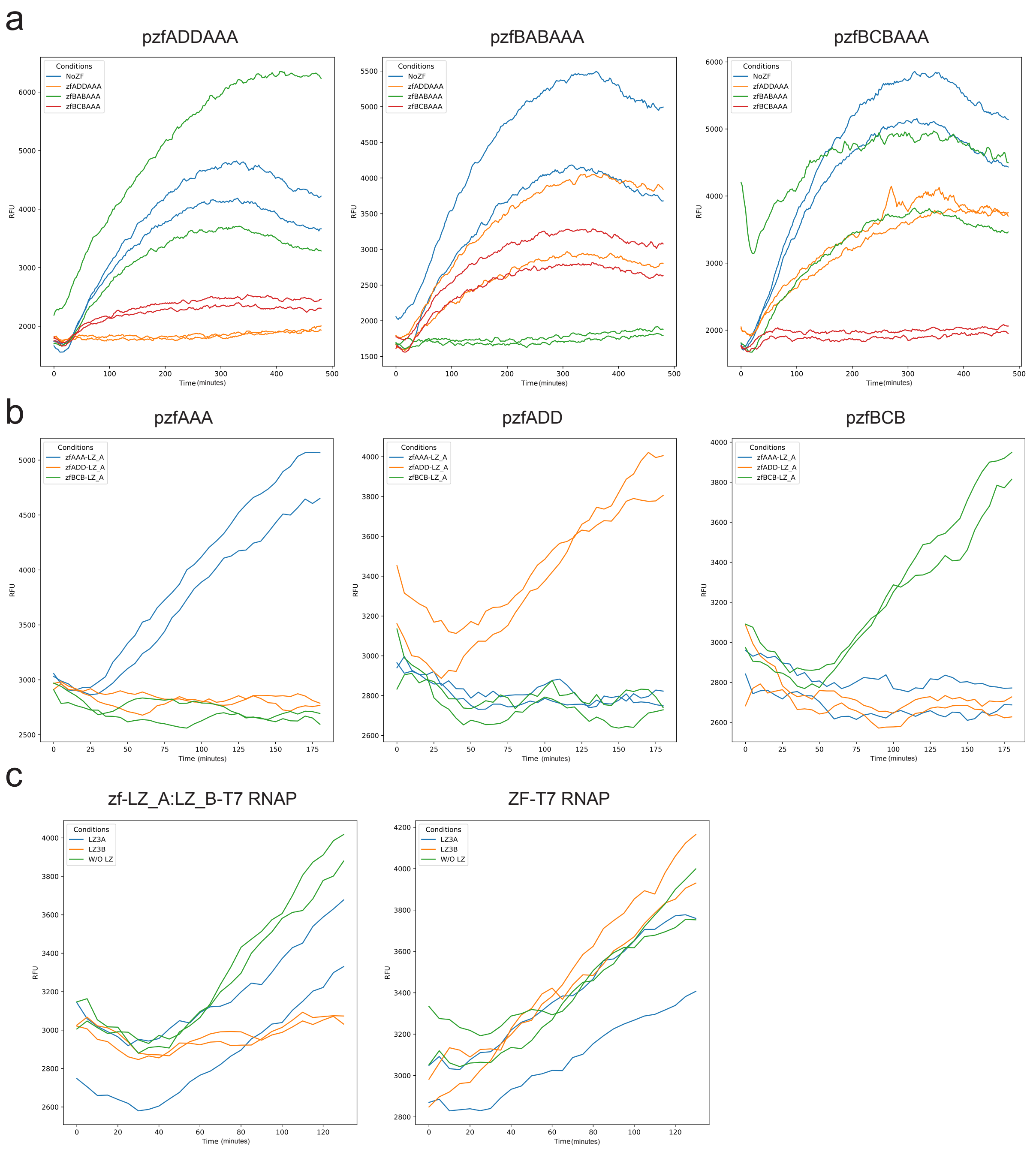

Supplementary Fig.4. Time-course transcription of regulatory component characterization: a, Programmable pzfxxxAAAd1T7 promoter repression dynamic (n=2). The curves were processed by moving average with the window size of 5, which applies to all panels in this figure. **b,** Programmable pzfXXXd1T7 promoter activation dynamic (n=2). **c,** LZ repression (n=2), left panel is the result from LZ system that is repressible by LZ, the right panel if ZF-T7 RNAP system that won't be influenced by LZ. Source data are provided as a Source Data file.

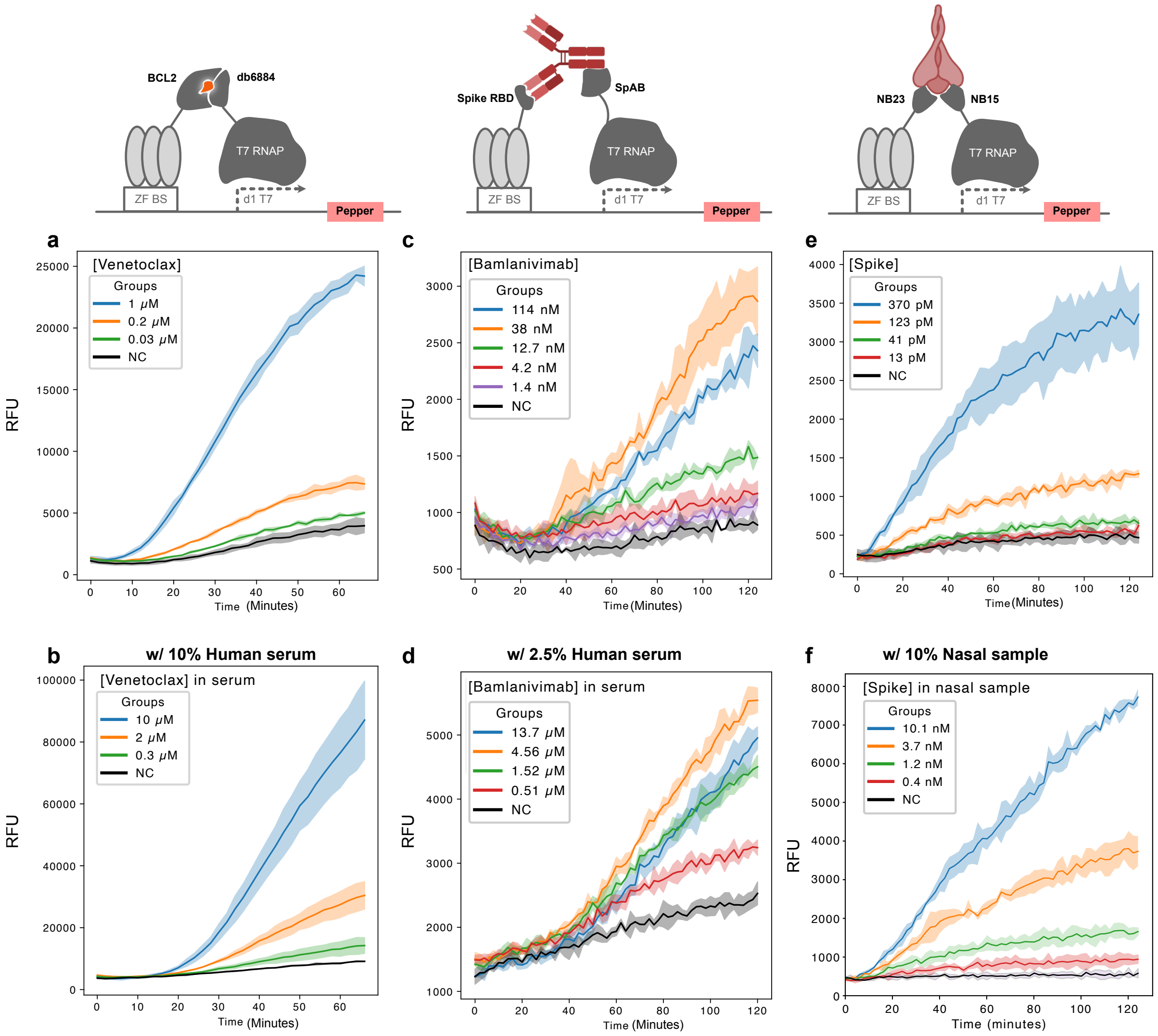

Supplementary Fig.5. Time-course of modular biomolecules sensor transcription:a, Venetoclax detection levels in a standard PURE reaction (n = 3; shaded regions indicate standard deviation; applies to all panels). **b,** Detection of Venetoclax in PURE with human serum. **c,** Detection of Bamlanivimab in a standard PURE reaction. **d,** Detection of Bamlanivimab in PURE with human serum. **e,** Detection of Spike in a IVT reaction. **f,** Detection of Spike in IVT with nasal swab sample. Source data are provided as a Source Data file.

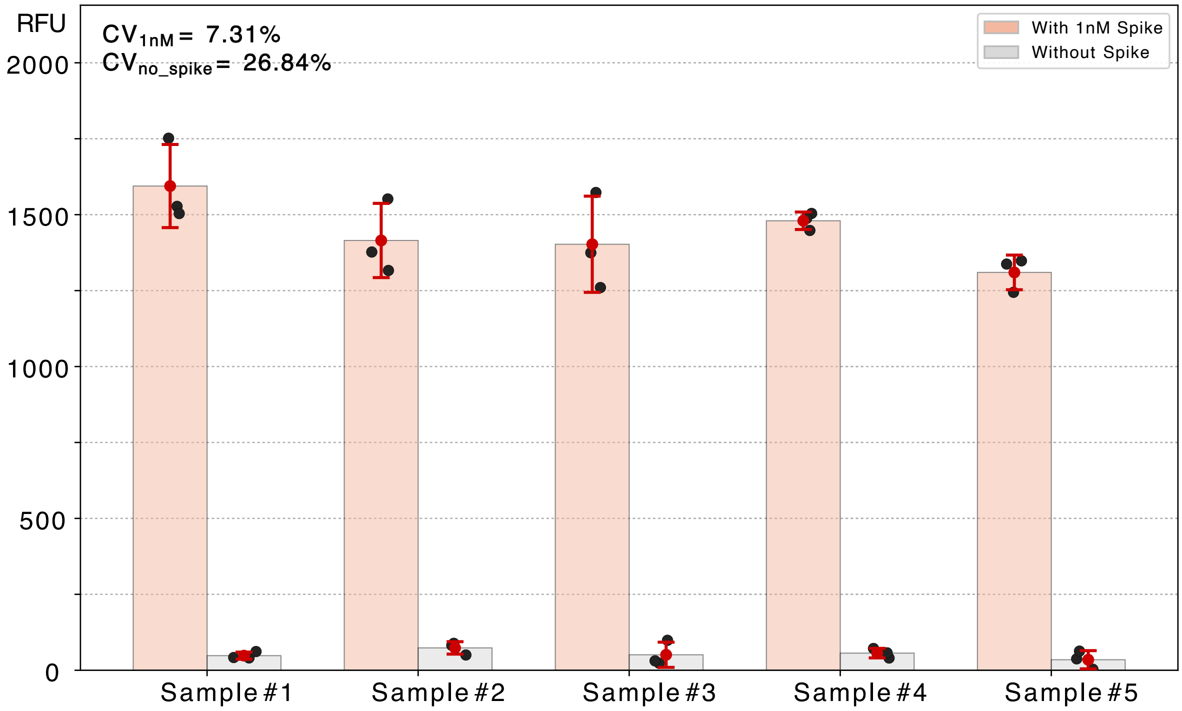

Supplementary Fig.6. Characterizing influence of different nasal samples on IVT reactions. Nasal samples from five individuals were collected to assess their effect on IVT reaction. Spike-sensing IVT reactions (n = 3) were performed with each nasal sample with 1 nM Spike protein or without Spike. The y-axis indicates the RFU yield of the Pepper reporter after 1 hour. Source data are provided as a Source Data file.

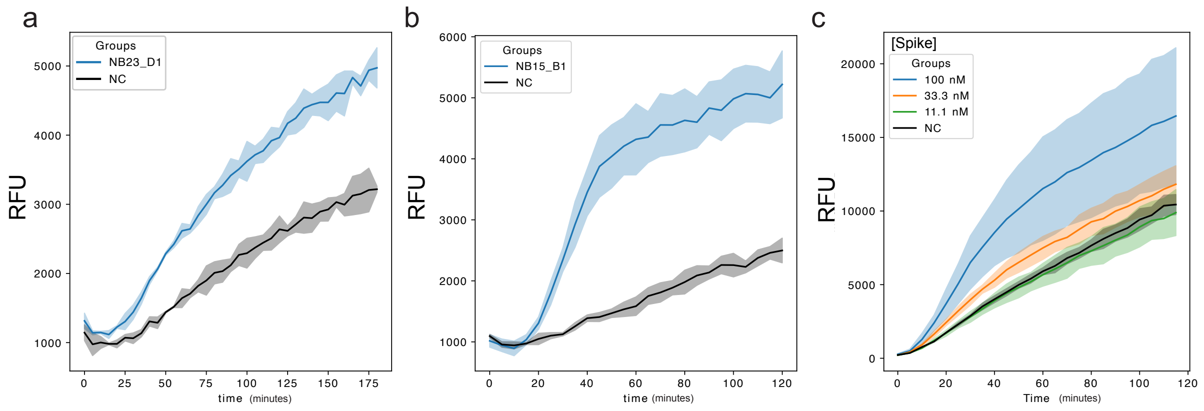

Supplementary Fig.7. Time-course of Spike detection reactions using de novo design binders:a, Validation of ZF-NB23_D1. **b,** Validation of ZF-NB15_B1. **c,** Spike detection with two de novo design binders complex NB23_D1 + NB15_B1- RNAP, demonstrating MDC of 33.3 nM. (n = 3; shaded regions indicate standard deviation; applies to all panels). Source data are provided as a Source Data file.

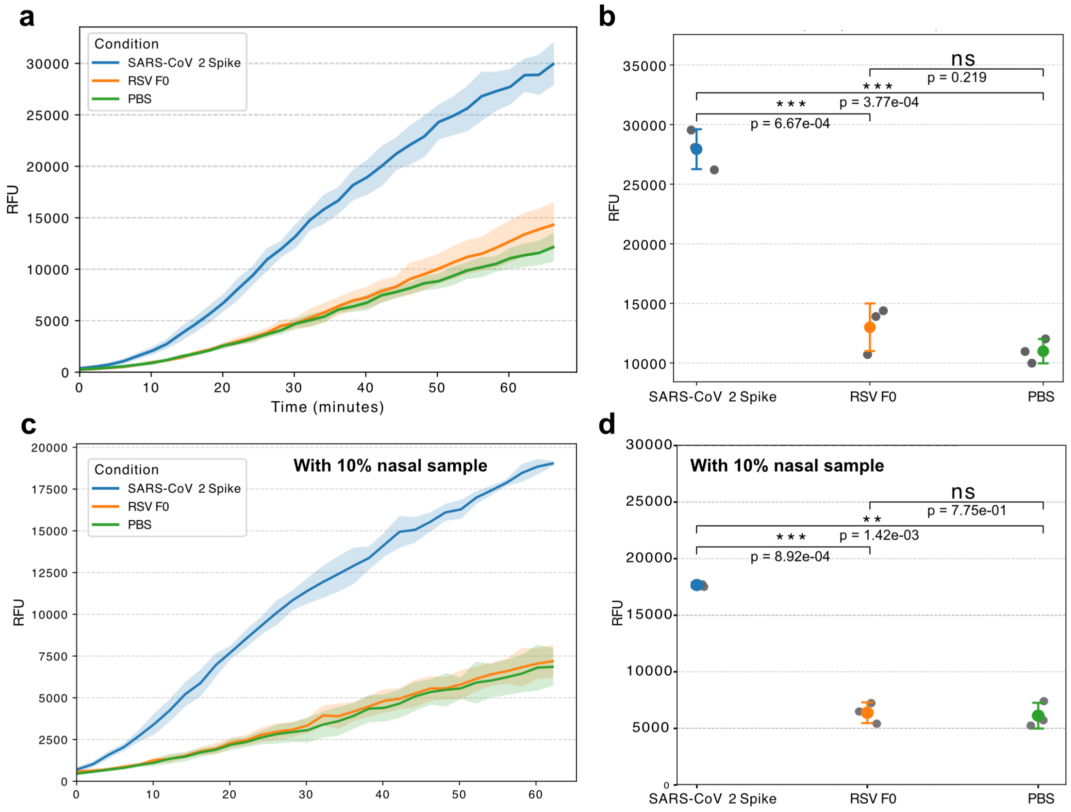

Supplementary Fig.8. Specificity of de novo–designed binders to Spike against RSV F0 protein.a, Time-course fluorescence readout comparing detection of 50 nM SARS-CoV-2 Spike and 50 nM RSV F0 proteins. (n = 3; shaded regions indicate standard deviation) **b,** Endpoint quantification (1 hour) and statistical analysis of fluorescence signals for SARS-CoV-2 Spike, RSV F0, and PBS control. Statistical significance is indicated as follows: ns, not significant; $^{**}p < 0.01$; $^{***}p < 0.001$. **c.**Time-course fluorescence readout in the presence of 10% nasal sample, comparing detection of 50 nM SARS-CoV-2 Spike and 50 nM RSV F0 proteins.(n = 3; shaded regions indicate standard deviation) **d.** Endpoint quantification (1 hour) and statistical analysis under 10% nasal sample conditions. Statistical significance is indicated as follows: ns, not significant; $^{**}p < 0.01$; $^{***}p < 0.001$. Source data are provided as a Source Data file.

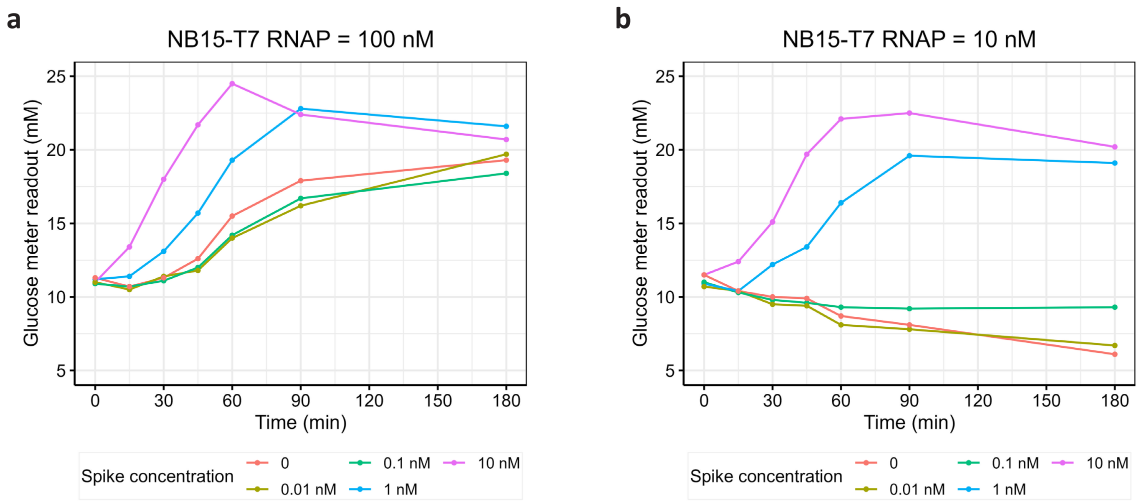

Supplementary Fig.9. Glucose signal over time for spike detection using △T7RNAP PURE with different NB15-T7RNAP concentrations: Glucose concentration was measured at different time intervals in samples with varying spike concentrations diluted into PBS. **a**, Reaction with 100 nM NB15-T7RNAP. **b,** Reaction with 10 nM NB15-T7RNAP. Each data point represents a single sample (n=1 applied to all panels). Source data are provided as a Source Data file.

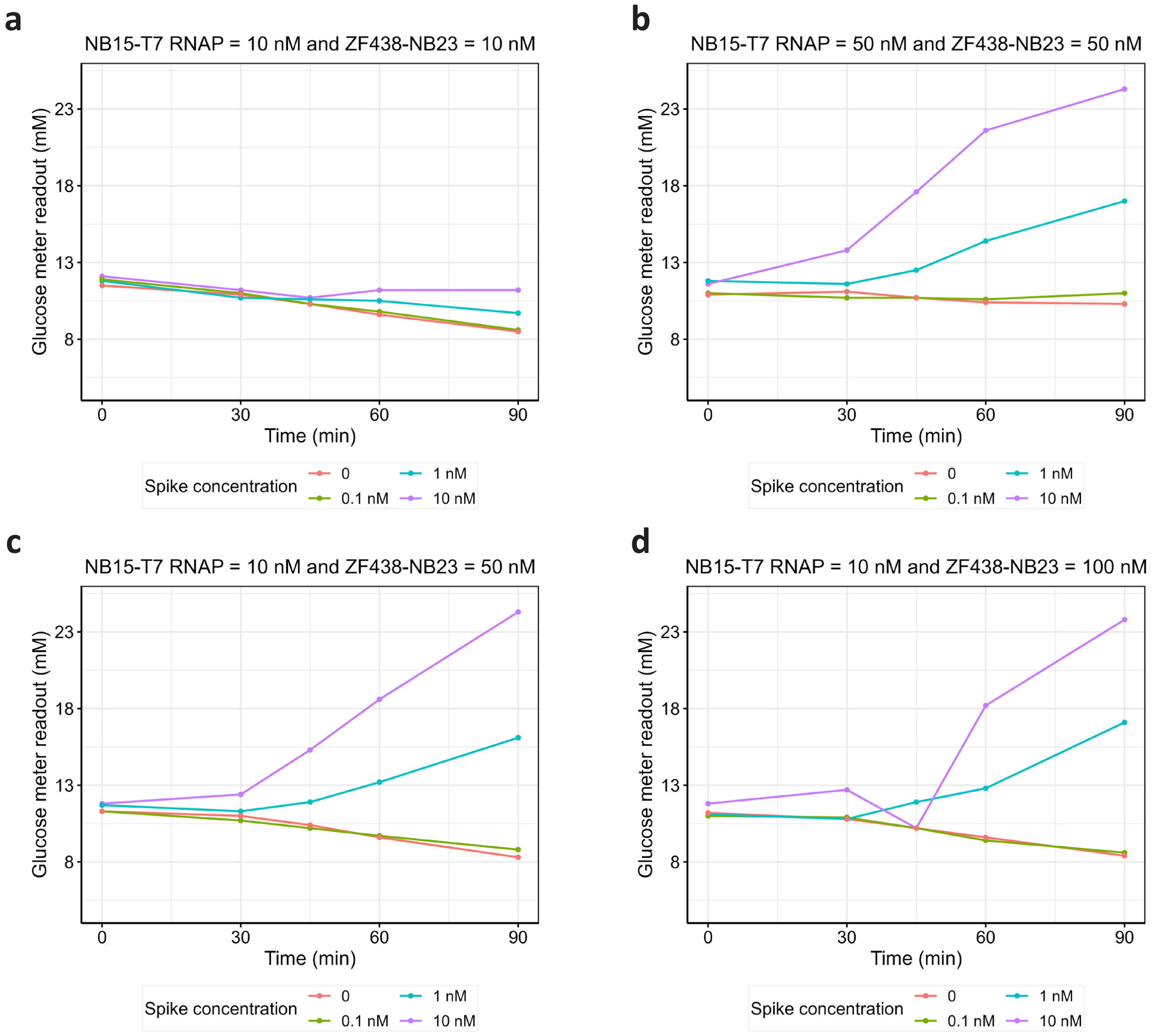

Supplementary Fig.10. Optimization of NB15-T7RNAP and ZF438-NB23 concentrations in ΔT7RNAP PURE for improved spike detection. Glucose concentration was measured at different time intervals in samples with varying spike concentrations(n=1). The concentrations of NB15-T7RNAP (N) and ZF438-NB23 (Z) in the different panels are as follows: **a,** N = 10 nM and Z = 10 nM, **b,** N = 50 nM and Z = 50 nM, **c,** N = 10 nM and Z = 50 nM, and **d,** N = 10 nM and Z = 100 nM (n=1 applied to all panels). Source data are provided as a Source Data file.

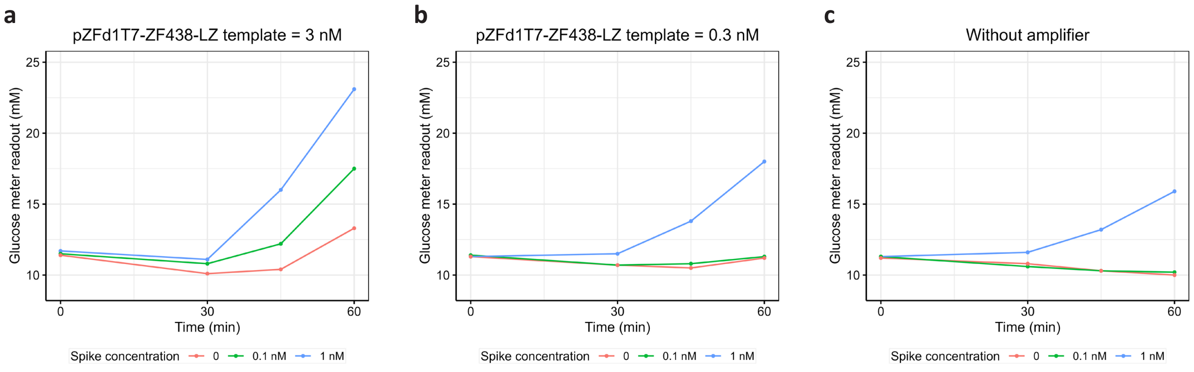

Supplementary Fig.11. Optimization of pZFd1T7-ZF438-LZ template concentration for the amplifier system. Glucose concentration was measured at different time intervals in samples with varying spike concentrations diluted into PBS (n =1). The pZFd1T7-ZF438-LZ template concentration is 3 nM in **a** and 0.3 nM in **b**. Panel **c** shows the same experiment without the amplifier.The concentrations of NB15-T7RNAP and ZF438-NB23 are the same in all samples (10 nM and 50 nM, respectively). T7RNAP-LZ is included in panels a and b at 10 nM (n=1 applied to all panels). Source data are provided as a Source Data file.

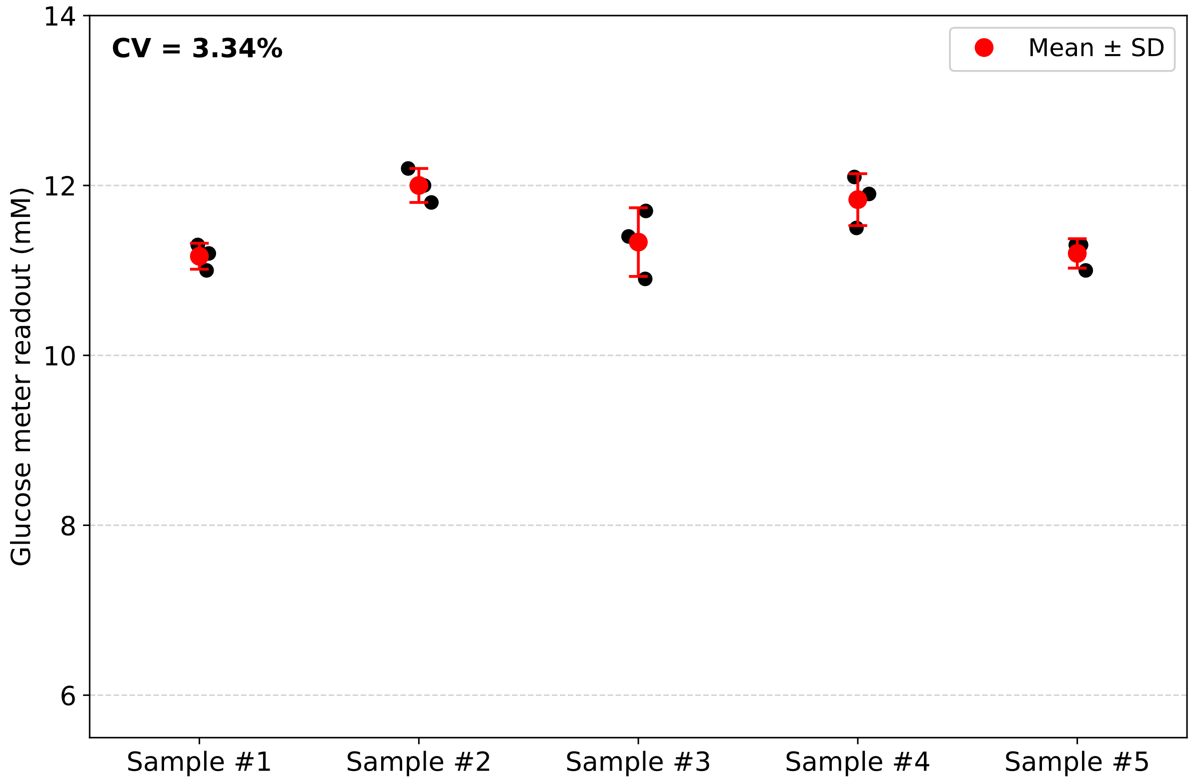

Supplementary Fig.12. Characterizing influence of different nasal samples PURE spike-sensing reactions with amplifier. Nasal samples from five individuals were collected to assess their effect on PURE-based sensing assays with amplifier circuit. Nasal samples with 1 nM Spike protein were added to PURE glucose readout reactions incorporating an amplifier circuit (n = 3). Glucose levels were quantified after 1 hour of incubation at 37 ℃. Source data are provided as a Source Data file.

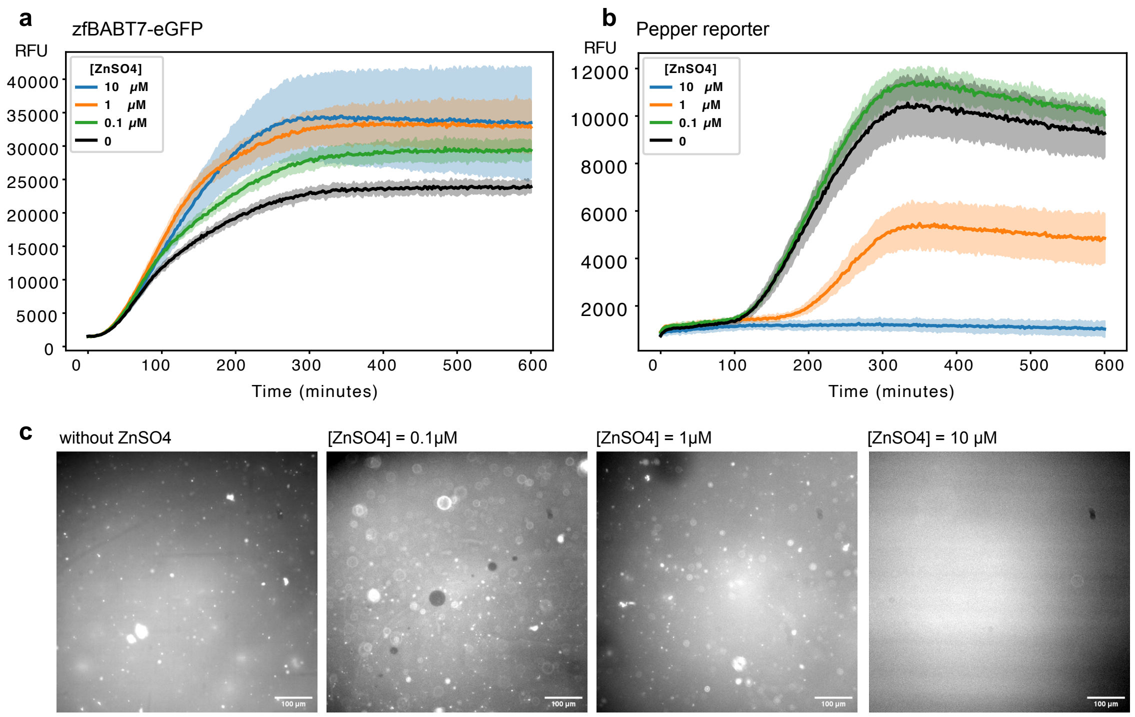

Supplementary Fig.13. Repression of pBABT7 promoter with zfBABT7 repressor under different zinc sulfate concentration. Reactions contained DNA templates encoding zfBABT7–eGFP and the pBABT7–pepper reporter. eGFP fluorescence was used to monitor repressor expression via plate reader and to assess aggregation by microscopy. **a,**Time-course eGFP fluorescence from zfBABT7–eGFP.(n = 3; shaded regions indicate standard deviation) **b,** Time-course Pepper reporter signal showing the characteristic de-repression phenotype at high repressor concentrations. De-repression is zinc-dependent: it occurs more slowly at 1 µM ZnSO4 and is not observed at 10 µM ZnSO4. (n = 3; shaded regions indicate standard deviation) **c,** Microscopy images of samples collected at the experimental endpoint. Aggregation of the repressor is observed in all conditions except 10 µM ZnSO4, consistent with the absence of de-repression in plate-reader measurements. Source data are provided as a Source Data file.

Linear template structure	5’common sequence - Promoter-5’UTR-RBS-ATG(start codon)-GOI- 3’UTR
5’common sequence	GCTACTGATTGATGACGTGC
5’UTR	ccacaacggtttccctctagaaataat
3’UTR	cggctgctaacaaagcccgaaaggaagctgagttggc tgctgccaccgctgagcaataactagcataaccccttggggcct ctaaacgggtcttgaggggttttttgctgaaaggaggaactata tcc
RBS	tttgtttaactttaagaaggagatatacc

Supplementary Table.1. DNA template structure and sequences. To design linear template for cell-free expression, follow the provided structure, then choose promoter and GOI sequence from following tables

Promoter name	Sequence
WT T7 promoter	TAATACGACTCACTATAGGGAGA
pADDT7	GCGGATGGAGTAATACGACTCACTATAGGGAGA
pBABT7	GAGTGGGTGGTAATACGACTCACTATAGGGAGA
pBCBT7	GAGGTAGTGGTAATACGACTCACTATAGGGAGA
pzf(ADDAAA)d1T7	TCCATCCGCCGCCCACGCtgaattgatcGCTAGCCGACTCACTATAGGGAGA
pzf(BABAAA)d1T7	CACCCACTCCGCCCACGCtgaattgatcGCTAGCCGACTCACTATAGGGAGA
pzf(BCBAAA)d1T7	CACTACCTCCGCCCACGCtgaattgatcGCTAGCCGACTCACTATAGGGAGA
pzf(AAA)d1T7	CGCCCACGCtgaattgatcGCTAGCCGACTCACTATAgggaga
pzf(ADD)d1T7	TCCATCCGCtgaattgatcGCTAGCCGACTCACTATAGGGAGA
pzf(BCB)d1T7	CACTACCTCtgaattgatcGCTAGCCGACTCACTATAgggaga
pzf(438)d1T7	gtcctcactcggaattgatcGCTAGCCGACTCACTATAGGGAGA
zf AAA binding site	GCGTGGGCG
zf ADD binding site	GCGGATGGA
zf BAB binding site	GAGTGGGTG
zf BCB binding site	GAGGTAGTG
zf T7 binding site	GTAATACGA

Supplementary Table.2. Promoter variants and ZF binding site sequence.

Name	Sequence
zfADDT7	MHHHHHHGGSLEPGEKPYKCPECGKSFSQSGHLTEHQRTHTGEKPYKCPECGKSFS<br>QKSSLIAHQRTHTGEKPYKCPECGKSFSQSSSLVRHQRTHRQKDGGGSERPYACPV<br>ESCDRRFSDKTKLRVHIRIHTGQKPFQCRICMRNFSVRHNLTRHIRTHTGEKPFACD<br>ICGRKFARSDERKRHTKIHLRQ
zfBABT7	MHHHHHHGGSLEPGEKPYKCPECGKSFSQSGHLTEHQRTHTGEKPYKCPECGKS<br>FSQKSSLIAHQRTHTGEKPYKCPECGKSFSQSSSLVRHQRTHRQKDGGGSERPY<br>ACPVESCDRRFSRNFILQRHIRIHTGQKPFQCRICMRNFSRSDHLTTHIRTHTG<br>EKPFACDICGRKFARHDQLTRHTKIHLRQ
zfBCBT7	MHHHHHHGGSLEPGEKPYKCPECGKSFSQSGHLTEHQRTHTGEKPYKCPECGKS<br>FSQKSSLIAHQRTHTGEKPYKCPECGKSFSQSSSLVRHQRTHRQKDGGGSERPY<br>ACPVESCDRRFSRNFILQRHIRIHTGQKPFQCRICMRNFSQRSSLVRHIRTHTG<br>EKPFACDICGRKFARHDQLTRHTKIHLRQ
zfADDAAA	MHHHHHHGGSERPYACPVESCDRRFSDKTKLRVHIRIHTGQKPFQCRICMRNFSV<br>RHNLTRHIRTHTGEKPFACDICGRKFARSDERKRHTKIHLRQKDGGGSERPYAC<br>PVESCDRRFSRSDELTRHIRIHTGQKPFQCRICMRNFSRSDHLTTHIRTHTGEKP<br>FACDICGRKFARSDERKRHTKIHLRQ
zfBABAAA	MHHHHHHGGSERPYACPVESCDRRFSRNFILQRHIRIHTGQKPFQCRICMRNFSR<br>SDHLTTHIRTHTGEKPFACDICGRKFARHDQLTRHTKIHLRQKDGGGSERPYACP<br>VESCDRRFSRSDELTRHIRIHTGQKPFQCRICMRNFSRSDHLTTHIRTHTGEKPF<br>ACDICGRKFARSDERKRHTKIHLRQ
zfBCBAAA	MHHHHHHGGSERPYACPVESCDRRFSRNFILQRHIRIHTGQKPFQCRICMRNFSQ<br>RSSLVRHIRTHTGEKPFACDICGRKFARHDQLTRHTKIHLRQKDGGGSERPYACP<br>VESCDRRFSRSDELTRHIRIHTGQKPFQCRICMRNFSRSDHLTTHIRTHTGEKPF<br>ACDICGRKFARSDERKRHTKIHLRQ
zfAAA-LZ_A	MERPYACPVESCDRRFSRSDELTRHIRIHTGQKPFQCRICMRNFSRSDHLTTHIR<br>THTGEKPFACDICGRKFARSDERKRHTKIHLRQEIAALEQEIAALEKENAALEWE<br>IAALEQ
zfADD-LZ_A	MERPYACPVESCDRRFSDKTKLRVHIRIHTGQKPFQCRICMRNFSVRHNLTRHIR<br>THTGEKPFACDICGRKFARSDERKRHTKIHLRQEIAALEQEIAALEKENAALEWE<br>IAALEQ
zfBCB-LZ_A	MERPYACPVESCDRRFSRNFILQRHIRIHTGQKPFQCRICMRNFSQRSSLVRHIRT<br>HTGEKPFACDICGRKFARHDQLTRHTKIHLRQEIAALEQEIAALEKENAALEWEIAA<br>LEQ
eGFP	VSKGEELFTGVVPILVELDGDVNGHKFSVSGEGEGDATYGKLTLKFICTTGKLP<br>VPWPTLVTTLTYGVQCFSRYPDHMKQHDFFKSAMPEGYVQERTIFFKDDGNYKTR<br>AEVKFEGDTLVNRIELKGIDFKEDGNILGHKLEYNYNSHNVYIMADKQKNGIKVN<br>FKIRHNIEDGSVQLADHYQQNTPIGDGPVLLPDNHYLSTQSALSKDPNEKRDHM<br>VLLEFVTAAGITLGMDELYK
Trehalase	MGTAVRIDYASGLTDRENSMFKEIQLSGVFADSKTFVDSHPKLPLAEIAELYHVR<br>QQQAGFDLAAFVHRYFELPPSIASGFVSDTSRPVEKHIDILWDVLTRQPDRQEAG<br>TLLPLPYPYVVPGGRFREIYYWDSYFTMLGLQASKRWDLMEGMVNNFSHLIDTIG<br>FIPNGNRTYYEGRSQPPFYALMVELLANKQGESVLLAHLPHLRREYEFWMEGAAKL<br>SPAAPAHRRVVLLPDGSILNRYWDDIAAPRPESFREDYELAEAIGGNKRELYRHIR<br>AAAESGWDFSSRWFKDGNGMASIHTTDIIPVDLNALVFNLERMLAHIYGLQGDQDQ<br>ATHYYQLAEQRKQALLRYCWNAQQGFFHDYDYVAAQQTPVMSLAAVYPLYFSMVDQ<br>RTGDRVAEQIEAHFIQAGGVTTTLATTGQQWDAPNGWAPLQWLTIQGLRNYHHNSA<br>AEQIKQRWIALNQRVYRNTGKLVEKYNVYDLDVAGGGGEYELQDGFGWTNGVLLHL<br>LNESTP
NB15	AQVQLVESGGGLVQAGGSLRLSCAASGRTFSSYAMGWFRQAPGKEREFVASINWNG<br>GNTYYADFVKGRFTISRDNAKNTVYLQMNSLKPEDTAVYYCAATGPNEYGLPREDL<br>FYDYWGQGTQVTVS
NB23	AQVHLVESGGDLVQPGGSLRLSCVASGSGFENNAITWYRQAPGKERELVSGITSG<br>GSTNYADSVKGRFTISRDNAKNTVYLEMNSLKPEDTAVYLCQAVAWDSRRRSVVA<br>FWGQGTQVTVS
NB23_D1	DKLKEKIKKLIQAIRDGASQDEIDKLLDEVAELGSGNLDLAILLNLITNLYLNG<br>YTFKEAEEAYKKVKAEKTTEEEKYLAEIYKNIKELLKKLGVDDDVF

Name	Sequence
NB15_C1	MATIEIDIKNEKTGRQAYLRYNATPENADLVIDNAEADIAAFAKLDPGNTVSIR GVADGGLEDLELARRAIERIEAAAKAAGVKVKSVELEASPSTQAAL
Spike_RBD	RVQPTESIVRFPNITNLCPFGEVFNATRFASVYAWNRKRISNCVADYSVLYNS ASFSTFKCYGVSPTKLNDLCFTNVYADSFVIRGDEVRQIAPGQTGKIADYNYK LPDDFTGCVIAWNSNNLDSKVGGNYNYLYRLFRKSNLKPFERDISTEIYQAGS TPCNGVEGFNCYFPLQSYGFQPTNGVGYQPYRVVVLSFELLHAPATVCGPKKS TNLVKNKCVNF
SpAB	ADNKFNKEQQNAFYEILHLPNLNEEQRNGFIQSLKDDPSQSANLLAEAKKLND AQAPK
BCL2	MAHAGRTGYDNREIVMKYIHYKLSQRGYEWDAGDDAEENRTEAPEGTESEVVHRA LRDAGDDFERRYRRDFAEMSSQLHLTPDTARQRFETVVEELFRDGVNWGRIVAFF EFGGVMCVESVNREMSPLVDNIAEWMTEYLNRHLHTWIQDNGGWDAFVELYGPSMR
DBVen1619	MQYLLVVKGPNVTKFRWVDSSSEAETLARKIAKKLGLEVKSVEKKGNAVRVEI
ZF438-GSGGG-Binder	FQCRICMRNFSRQDRLDRHTRTHTGEKPFQCRICMRNFSQKEHLAGHLRTHTG EKPFQCRICMRNFSRRDNLNRHLKTHGSGGG-Binder
Binder - T7 RNAP	MHHHHHH - Binder- GSGGGGSGGGGSGGGGSMNTINIAKNDFSDIELAAIPFNTLADHYGERLAR EQLALEHESYEMGEARFRKMFERQLKAGEVADNAAAKPLITTLLPKMIAR INDWFEEVKAKRGKRPTAFQFLQEIKPEAVAYITIKTTLACLTSADNTTVQAV ASAIGRAIEDEARFGRIRDLEAKHFKKNVEEQLNKRVGHVYKKAFMQVVEA DMLSKGLLGGEAWSSWHKEDSIHVGVRCIEMLIESTGMVSLHRQNAGVVGQ DSETIELAPEYAEAIATRAGALAGISPMFQPCVVPPKPWTGITGGGYWANGRR PLALVRTHSKKALMRYEDVYMPEVYKAINIAQNTAWKINKKVLAVANVITKW KHCPVEDIPAIEREELPMKPEDIDMNPEALTAWKRAAAAVYRKDKARKSRRI SLEFMLEQANKFANHKAIWFPYNMDWRGRVYAVSMFNPQGNDMTKGLLT LAKGKPIGKEGYYWLKIHGANCAGVDKVPFPERIKFIEENHENIMACAKSPLE NTWWAEQDSPFCFLAFCFEYAGVQHHGLSYNCSLPLAFDGSCSGIQHFSAMLR DEVGGRAVNLLPSETVQDIYGIVAKKVNEILQADAINGTDNEVVTVTDENTGEIS EKVKLGTKALAGQWLAYGVTRSVTKRSVMTLAYGSKEFGFRQQVLEDTIQPAIDS GKGLMFTQPNQAAGYMAKLIWESVSVTVVAAVEAMNWLKSAAKLLAAEVK DKKTGEILRKRCAVHWVTPDGFPVWQEYKKPIQTRLNLMFLGQFRLQPTINTN KDSEIDAHKQESGIAPNFVHSQDGSHLRKTVVWAHEKYGIESFALIHDSFGTIPA DAANLFKAVRETMVDTYESCDVLADFYDQFADQLHESQLDKMPALPAKGNLNL RDILESDFAFA
Broccoli_aptamer	ttgccatgtgtatgtgggagacggtcgggtccagatattcgtatctgtcgagtagagtgtgggctcccacat actctgatgatccttcgggatcattcatggcaa
Pepper_aptamer	TTGCCATGTGTATGTGGGTTCGCCCACATACTCTGATGATCCCCAATCGTGGC GTGTCGGCCTGCTTCGGCAGGCACTGGCGCCGGGATCATTCATGGCAA

Supplementary Table.3. Sequence of proteins and reporters.

Components	Buffer A	Buffer B	Buffer HT	Stock buffer A	Stock buffer B
HEPES	50 mM	50 mM	50 mM	50 mM	50 mM
Ammonium chloride	1000 mM	-	-	-	-
Magnesium chloride	10 mM	10 mM	10 mM	10 mM	10 mM
Potassium chloride	-	100 mM	100 mM	100 mM	100 mM
Imidazole (pH=7)	-	500 mM	-	-	-
Glycerol	-	-	-	30% (v/v)	60% (v/v)
TCEP	1 mM	1 mM	1 mM	1 mM	1 mM

Supplementary Table.4. Protein purification Buffers.

Components	Without serum	With serum
PURExpress Solution A	4 µL	4 µL
PURExpress Solution B	3 µL	3 µL
HBC620	10 µM	10 µM
Pre-expressed zf438-BCL2 (5x diluted)	0.25 µl	0.25 µl
DBven_1619-T7RNAP	100 nM	100 nM
Human serum	–	1 µl
NEB RNAse Inhibitor	–	1U/µl
Venetoclax	0.5 µl	0.5 µl
DNA Template: pZF438d1T7-Pepper	10 nM	10 nM
**Total Volume**	**10 uL**	**10 uL**

Supplementary Table.5. Venetoclax sensing PURE reaction composition.

Components	Without serum	With serum
PURExpress Solution A	4 µL	4 µL
PURExpress Solution B	3 µL	3 µL
HBC620	10 µM	10 µM
Pre-expressed zf438-RBD (2x diluted)	0.1 µl	0.1 µl
SpAB-T7RNAP	100 nM	400 nM
Human serum	–	0.25 µl
NEB RNAse Inhibitor	–	1U/µl
NEB Disulfide bond enhancer 1	0.4µl	0.4µl
NEB Disulfide bond enhancer 2	0.4 µl	0.4 µl
Bamlanivimab	0.5 µl	0.5 µl
DNA Template: pZF438d1T7-Pepper	10 nM	10 nM
**Total Volume**	**10 uL**	**10 uL**

Supplementary Table.6. Antibody sensing PURE reaction composition.

Components	Concentration
HEPES	250 mM
Potassium glutamate	500 mM
Magnesium acetate	59 mM
ATP	5 mM
GTP	5 mM
CTP	5 mM
UTP	5 mM
TCEP	5 mM
Spermidine	10 mM

Supplementary Table.7. 5x IVT buffer.

Components	Without nasal sample	With nasal sample
5x IVT buffer	4 µL	4 µL
HBC620	10 µM	10 µM
ZF438-NB23	80 nM	80 nM
NB15-T7RNAP	3.3 nM	3.3 nM
Nasal sample	–	2 µl
NEB RNAse Inhibitor	–	1U/µl
Spike protein	1 µl	1 µl
DNA Template: pZF438d1T7-Pepper	10 nM	10 nM
**Total Volume**	**20 uL**	**20 uL**

Supplementary Table.8. Spike sensing IVT reaction composition.

Components	
5x IVT buffer	4 µL
HBC620	10 µM
DFHBI-1T	10 µM
ZF438-NB23	10 nM
ZFAAA-BCL2	30 nM
NB15-T7RNAP	10 nM
DBven_1619-T7RNAP	20 nM
Venetoclax	1 µl
Spike protein	1 µl
DNA Template: pZF438d1T7-Pepper	10 nM
DNA Template: pZFAAAd1T7-Broccoli	10 nM
**Total Volume**	**20 uL**

Supplementary Table.9. Multiplexed Spike and Venetoclax sensing IVT reaction composition.

Components	volume or concentration
PURExpress Solution A	4 µL
PURExpress Solution B	3 µL
Spike protein	10 nM
ZF-binder DNA template	100 pg (17.5 pM- 19.1 pM based on DNA length)
NB15-T7RNAP or NB23-T7 RNAP	10 nM
HBC 620	10 µM
Reporter DNA template	10 nM
**Total Volume**	**10 uL**

Supplementary Table.10. Composition of the cell-free reaction for de novo design binder screening.

Number	Protein	Protein Name	Concentration in Reaction [µg/ml]
1	AlaRS	Alanyl-tRNA synthetase	70.0
2	ArgRS	Arginyl-tRNA synthetase	2.0
3	AsnRS	Asparaginyl-tRNA synthetase	22.0
4	AspRS	Aspartate-tRNA synthetase	8.0
5	CysRS	Cysteinyl-tRNA synthetase	1.2
6	GlnRS	Glutaminyl-tRNA synthetase	3.8
7	GluRS	Glutamyl-tRNA synthetase	12.6
8	GlyRS	Glycyl-tRNA synthetase	9.6
9	HisRS	Histidyl-tRNA synthetase	0.8
10	IleRS	Isoleucyl-tRNA synthetase	40.0
11	LeuRS	Leucyl-tRNA synthetase	4.0
12	LysRS	Lysyl-tRNA synthetase	6.4
13	MetRS	Methionine-tRNA synthetase	2.3
14	PheRS	Phenylalanyl-tRNA synthetase	17.0
15	ProRS	Prolyl-tRNA synthetase	10.0
16	SerRS	Seryl-tRNA synthetase	1.9
17	ThrRS	Threonyl-tRNA synthetase	6.2
18	TrpRS	Tryptophanyl-tRNA synthetase	6.3
19	TyrRS	Tyrosyl-tRNA synthetase	0.6
20	ValRS	Valyl-tRNA synthetase	1.8
21	IF1	Initiation factor 1	1.0
22	IF2	Initiation factor 2	4.0
23	IF3	Initiation factor 3	10.0
24	EF-G	Elongation factor G	50.0
25	EF-Tu	Elongation factor Tu	500.0
26	EF-Ts	Elongation factor Ts	50.0
27	RF1	Release factor 1	10.0
28	RF2	Release factor 2	10.0
29	RF3	Release factor 3	10.0
30	RRF	Ribosome recycling factor	10.0
31	MTF	Methionyl-tRNA formyltransferase	20.0
32	CK	Creatine kinase	4.0
33	MK	Adenylate kinase (Myokinase)	3.0
34	NDK	Nucleotide diphosphate kinase	1.1
35	PPiase	Inorganic pyrophosphatase	1.0
36	T7 RNAP*	T7 RNA polymerase	10.0

Supplementary Table.11. Final concentration of PURE Proteins in the Reaction.
*For the homemade ΔT7 RNAP PURE protein mixture, T7 RNAP was excluded.

Components	Without Amplifier	With Amplifier
PURExpress Solution A	4 µL	4 µL
Homemade ∆T7RNAP PURE	1.3 µL	1.3 µL
NEB Ribosome	0.45 µL	0.45 µL
ZF438-NB23	50 nM	50 nM
NB15-T7RNAP	10 nM	10 nM
LZ_B-T7RNAP	–	10 nM
NEB RNase Inhibitor	1U/µl	1U/µl
Trehalose	10 mM	10 mM
DNA Template: pZFd1T7-ZF-LZ_A	–	3 nM
DNA Template: pZFd1T7-Tre37A	20 nM	20 nM
**Total Volume**	**10 uL**	**10 uL**

Supplementary Table.12. Composition of the cell-free reaction for spike detection via glucose generation with and without amplifier.

Components	Without Amplifier	With Amplifier
PURExpress Solution A	4 uL	4 uL
Homemade ∆T7RNAP PURE	1.3 uL	1.3 uL
NEB Ribosome	0.45 uL	0.45 uL
ZF438-NB23	50 nM	50 nM
NB15-T7RNAP	10 nM	10 nM
LZ_B-T7RNAP	-	10 nM
NEB RNase Inhibitor	1U/µl	1U/µl
HBC620	10 nM	10 nM
DNA Template: pZFd1T7-ZF-LZ_A	-	1 nM
DNA Template: pZFd1T7-Pepper	6 nM	6 nM
**Total Volume**	**10 uL**	**10 uL**

Supplementary Table.13. Composition of the cell-free reaction for spike detection using the Pepper aptamer, with and without amplifier.
