# Supplementary material for: A T7 RNAP regulatory toolbox for cell-free network engineering and biosensing applications
Source: Nat Commun. 2026 May 28;17:6941. doi: 10.1038/s41467-026-73811-9 (PMC13389190; doi:10.1038/s41467-026-73811-9)
Supplement: Supplementary file 2 — Description of Additional Supplementary Information [file 41467_2026_73811_MOESM2_ESM.pdf]

Description of Additional Supplementary Information

Supplementary Data 1 : "NB15 de novo binder from RFdiffusion" lists all designs generated by the RFdiffusion pipeline, and "NB15 screened ZF-binder DNA sequence" provides the selected binders and corresponding DNA sequences ordered for testing. Similarly, "NB23 de novo binder from RFdiffusion" lists all designs generated by the RFdiffusion pipeline, and "NB23 screened ZF-binder DNA sequence" provides the selected binders and corresponding DNA sequences ordered for testing.
