# Supplementary material for: A T7 RNAP regulatory toolbox for cell-free network engineering and biosensing applications
Source: Nat Commun. 2026 May 28;17:6941. doi: 10.1038/s41467-026-73811-9 (PMC13389190; doi:10.1038/s41467-026-73811-9)
Supplement: Supplementary file 4 — Reporting Summary [file 41467_2026_73811_MOESM4_ESM.pdf]

nature portfolio

Corresponding author(s): Sebastian Maerkl

Last updated by author(s): May 20, 2026

Reporting Summary

Nature Portfolio wishes to improve the reproducibility of the work that we publish. This form provides structure for consistency and transparency in reporting. For further information on Nature Portfolio policies, see our Editorial Policies and the Editorial Policy Checklist.

Statistics

For all statistical analyses, confirm that the following items are present in the figure legend, table legend, main text, or Methods section.

n/a	Confirmed	
☐	☒	The exact sample size (n) for each experimental group/condition, given as a discrete number and unit of measurement
☐	☒	A statement on whether measurements were taken from distinct samples or whether the same sample was measured repeatedly
☐	☒	The statistical test(s) used AND whether they are one- or two-sided<br>*Only common tests should be described solely by name; describe more complex techniques in the Methods section.*
☒	☐	A description of all covariates tested
☒	☐	A description of any assumptions or corrections, such as tests of normality and adjustment for multiple comparisons
☒	☐	A full description of the statistical parameters including central tendency (e.g. means) or other basic estimates (e.g. regression coefficient) AND variation (e.g. standard deviation) or associated estimates of uncertainty (e.g. confidence intervals)
☐	☒	For null hypothesis testing, the test statistic (e.g. F, t, r) with confidence intervals, effect sizes, degrees of freedom and P value noted<br>*Give P values as exact values whenever suitable.*
☒	☐	For Bayesian analysis, information on the choice of priors and Markov chain Monte Carlo settings
☒	☐	For hierarchical and complex designs, identification of the appropriate level for tests and full reporting of outcomes
☒	☐	Estimates of effect sizes (e.g. Cohen's d, Pearson's r), indicating how they were calculated

Our web collection on statistics for biologists contains articles on many of the points above.

Software and code

Policy information about availability of computer code

Data collection	*Provide a description of all commercial, open source and custom code used to collect the data in this study, specifying the version used OR state that no software was used.*
Data analysis	data is analyzed with python scipy.stats package.

For manuscripts utilizing custom algorithms or software that are central to the research but not yet described in published literature, software must be made available to editors and reviewers. We strongly encourage code deposition in a community repository (e.g. GitHub). See the Nature Portfolio guidelines for submitting code & software for further information.

Data

Policy information about availability of data

All manuscripts must include a data availability statement. This statement should provide the following information, where applicable:

- Accession codes, unique identifiers, or web links for publicly available datasets
- A description of any restrictions on data availability
- For clinical datasets or third party data, please ensure that the statement adheres to our policy

The dataset of this manuscript is avaialable on figshare : https://doi.org/10.6084/m9.figshare.32243667

Research involving human participants, their data, or biological material

Policy information about studies with human participants or human data. See also policy information about sex, gender (identity/presentation), and sexual orientation and race, ethnicity and racism.

Reporting on sex and gender	The information of sex and gender are not recorded while acquiring the nasal sample.
Reporting on race, ethnicity, or other socially relevant groupings	The information of race and ethnicity are not recorded while acquiring the nasal sample.
Population characteristics	The information of population characteristics are not recorded while acquiring the nasal sample.
Recruitment	Participants are recruited withing EPFL.
Ethics oversight	The study protocol is approved by EPFL Human Research Ethics Committee (HREC)

Note that full information on the approval of the study protocol must also be provided in the manuscript.

Field-specific reporting

Please select the one below that is the best fit for your research. If you are not sure, read the appropriate sections before making your selection.

☒ Life sciences ☐ Behavioural & social sciences ☐ Ecological, evolutionary & environmental sciences

For a reference copy of the document with all sections, see nature.com/documents/nr-reporting-summary-flat.pdf

Life sciences study design

All studies must disclose on these points even when the disclosure is negative.

Sample size	For experiments characterizing inter-participant variation in nasal samples (Supplementary Figs. 6 and 12), samples from five individuals were analyzed. Five participants were considered sufficient to evaluate sample variability and address the reviewers' concerns. For each sample, measurements were performed in triplicate using independently prepared reactions.<br><br>For the results shown in Fig. 3, a single nasal sample was used and the analyte concentration was titrated. Each analyte concentration was measured in triplicate using independently prepared reactions.
Data exclusions	no data exclusion
Replication	For experiments with replicates, each reaction was prepared independently and measured in a separate well of the plate. Results from replicates were collected within the same experiment.<br><br>For experiments comparing experimental conditions with controls (Figs. 3, 4, and 5), each condition was measured in three independent replicates, which were considered sufficient to evaluate the statistical significance of differences between conditions and controls.<br><br>Characterization of regulatory components (activation and repression behavior) was performed using duplicate measurements, as these experiments were intended to assess regulatory behavior rather than support statistical analysis.<br><br>De novo binder screening (Fig. 4b,c) and the glucose-readout condition screen were performed as single measurements. In the binder screen experiment, top-performing candidates were subsequently validated in triplicate. For the glucose diagnostic condition screen, a titration gradient provided sufficient resolution to identify optimal conditions without replication.
Randomization	no randomization
Blinding	no blinding

Reporting for specific materials, systems and methods

We require information from authors about some types of materials, experimental systems and methods used in many studies. Here, indicate whether each material, system or method listed is relevant to your study. If you are not sure if a list item applies to your research, read the appropriate section before selecting a response.

Materials & experimental systems

n/a	Involved in the study
☐	☒ Antibodies
☒	☐ Eukaryotic cell lines
☒	☐ Palaeontology and archaeology
☒	☐ Animals and other organisms
☒	☐ Clinical data
☒	☐ Dual use research of concern
☒	☐ Plants

Methods

n/a	Involved in the study
☒	☐ ChIP-seq
☒	☐ Flow cytometry
☒	☐ MRI-based neuroimaging

Antibodies

Antibodies used	Bamlanivimab
Validation	the antibody is from Lucerna-Chem, cat no HY-P9934401MG

Plants

Seed stocks	*Report on the source of all seed stocks or other plant material used. If applicable, state the seed stock centre and catalogue number. If plant specimens were collected from the field, describe the collection location, date and sampling procedures.*
Novel plant genotypes	*Describe the methods by which all novel plant genotypes were produced. This includes those generated by transgenic approaches, gene editing, chemical/radiation-based mutagenesis and hybridization. For transgenic lines, describe the transformation method, the number of independent lines analyzed and the generation upon which experiments were performed. For gene-edited lines, describe the editor used, the endogenous sequence targeted for editing, the targeting guide RNA sequence (if applicable) and how the editor was applied.*
Authentication	*Describe any authentication procedures for each seed stock used or novel genotype generated. Describe any experiments used to assess the effect of a mutation and, where applicable, how potential secondary effects (e.g. second site T-DNA insertions, mosiacism, off-target gene editing) were examined.*
