# Supplementary material for: A T7 RNAP regulatory toolbox for cell-free network engineering and biosensing applications
Source: Nat Commun. 2026 May 28;17:6941. doi: 10.1038/s41467-026-73811-9 (PMC13389190; doi:10.1038/s41467-026-73811-9)
Supplement: Supplementary file 5 — Transparent Peer Review file [file 41467_2026_73811_MOESM5_ESM.pdf]

nature portfolio

Peer Review File

A T7 RNAP regulatory toolbox for cell-free network engineering and biosensing applications

Corresponding Author: Professor Sebastian Maerkl

This file contains all reviewer reports in order by version, followed by all author rebuttals in order by version.

Version 1:

Reviewer comments:

Reviewer #1

(Remarks to the Author)
The authors have thoroughly revised the manuscript and successfully addressed all of my previous comments and suggestions. The revisions are comprehensive, and all changes have been implemented. The updated version shows clear improvement in clarity, structure, and scientific rigor. The responses are detailed and clear, I appreciate the authors' thoughtful engagement with the feedback.

The only, and biggest, criticism I have is about the novelty.
The authors explicitly state that their system is based on d1T7 published in 2018 and describe it as an "old sensing framework." Improvements built on an existing platform, even substantial performance gains, do not constitute conceptual novelty unless they arise from a fundamentally new principle or design. The manuscript itself confirms that the central mechanism relies on truncated T7 RNAP variants (d1T7), DNA-binding domain–guided recruitment and ligand-induced dimerization to control transcription. These strategies were already demonstrated prior to this work. Adapting them to new targets or contexts represents optimization and application, not invention of a new regulatory paradigm.
The rebuttal claims three orders of magnitude improvement in sensitivity compared to a split-T7 system. Performance improvements alone do not establish novelty. In this case, the (impressive!) performance improvement arises from optimization efforts, not novel engineering. None of the methods used to improve performance in this work require new mechanistic insight.
The paper itself emphasizes system engineering within a cell-free context rather than introducing a new transcriptional control principle. Sensitivity improvements are best interpreted as engineering refinement, not conceptual advance.
I think this is an interesting paper and a good tool for the community, but to avoid overstatement it should be framed as tool engineering, removing claims of novelty.

I strongly believe it's worth publishing and the paper's technical aspects are improved in revisions.

Reviewer #3

(Remarks to the Author)
Dear Authors,

Thank you for your detailed and thoughtful responses to our comments, as well as for the substantial revisions made to the manuscript. We appreciate the care with which you addressed both the conceptual and technical concerns.

We find that the revised manuscript is significantly improved. In particular:
- The authors have added new experimental data to support claims regarding multiplexing, matrix stability, and quantitative comparisons to wt T7RNAP.
- Methodological clarity has been significantly improved, including clearer reporting of concentrations, additional details on PURE system preparation, and expanded explanations in the methods section.
- Several conceptual criticisms regarding novelty, sensitivity, and applicability have been addressed through additional data and expanded discussion. Most significantly, the authors have clarified how their approach differs from prior split T7 approaches and outlined key advancements from the published d1T7 system.

- Figures have been revised for clarity, and terminology (e.g. LOD vs. MDC) has been corrected where appropriate.

While some limitations remain (e.g. lack of clarity if Venetoclax could be monitored at home using minimally invasive blood collection and reliance on recombinant spike rather than virions), the revisions substantially improve transparency and technical rigor compared to the original submission.

Major concern:
The authors failed to adequately address Reviewer 1's concern, regarding "The paper presents de novo design of functional protein binders (NB23 D1 and NB15 C1) using RFdiffusion, but there is no experimental binding data". Specificity is a critical issue in antibody engineering, particularly for weak binders, and without proper ELISA data it is difficult to conclude that these nanobodies are truly specific for the spike protein. Nanobodies should be tested against both the spike protein and a non-specific control antigen, alongside a non-specific antibody or nanobody as a negative control. The author's claim that "Our experiments show that signal is only generated in response to the addition of spike protein, our binders therefore do recognize and bind to spike." is not sufficient.

At this stage, if the authors can provide experimental evidence to support the specificity of their computationally generated nanobodies, we will recommend publication in Nature Communications.

Reviewer #4

(Remarks to the Author)
I co-reviewed this manuscript with one of the reviewers who provided the listed reports. This is part of the Nature Communications initiative to facilitate training in peer review and to provide appropriate recognition for Early Career Researchers who co-review manuscripts.

Open Access This Peer Review File is licensed under a Creative Commons Attribution 4.0 International License, which permits use, sharing, adaptation, distribution and reproduction in any medium or format, as long as you give appropriate credit to the original author(s) and the source, provide a link to the Creative Commons license, and indicate if changes were made.
In cases where reviewers are anonymous, credit should be given to 'Anonymous Referee' and the source.
The images or other third party material in this Peer Review File are included in the article's Creative Commons license, unless indicated otherwise in a credit line to the material. If material is not included in the article's Creative Commons license and your intended use is not permitted by statutory regulation or exceeds the permitted use, you will need to obtain permission directly from the copyright holder.
To view a copy of this license, visit https://creativecommons.org/licenses/by/4.0/

We thank reviewers for their additional insightful and helpful comments for improving this manuscript. We conducted additional experiments, and addressed the remaining comments in this rebuttal letter and revised the manuscript accordingly. Responses to all reviewer comments are highlighted in blue.

REVIEWER COMMENTS

Reviewer #1 (Remarks to the Author): The authors have thoroughly revised the manuscript and successfully addressed all of my previous comments and suggestions. The revisions are comprehensive, and all changes have been implemented. The updated version shows clear improvement in clarity, structure, and scientific rigor. The responses are detailed and clear, I appreciate the authors' thoughtful engagement with the feedback.

The only, and biggest, criticism I have is about the novelty. The authors explicitly state that their system is based on d1T7 published in 2018 and describe it as an "old sensing framework." Improvements built on an existing platform, even substantial performance gains, do not constitute conceptual novelty unless they arise from a fundamentally new principle or design. The manuscript itself confirms that the central mechanism relies on truncated T7 RNAP variants (d1T7), DNA-binding domain–guided recruitment and ligand-induced dimerization to control transcription. These strategies were already demonstrated prior to this work. Adapting them to new targets or contexts represents optimization and application, not invention of a new regulatory paradigm. The rebuttal claims three orders of magnitude improvement in sensitivity compared to a split-T7 system. Performance improvements alone do not establish novelty. In this case, the (impressive!) performance improvement arises from optimization efforts, not novel engineering. None of the methods used to improve performance in this work require new mechanistic insight. The paper itself emphasizes system engineering within a cell-free context rather than introducing a new transcriptional control principle. Sensitivity improvements are best interpreted as engineering refinement, not conceptual advance. I think this is an interesting paper and a good tool for the community, but to avoid overstatement it should be framed as tool engineering, removing claims of novelty.

I strongly believe it's worth publishing and the paper's technical aspects are improved in revisions.

We thank the reviewer for raising this important point regarding novelty. We agree that our work builds on the previously described d1T7 system by Hussey et al., and we have revised the manuscript to more clearly reflect this foundation and avoid overstating conceptual novelty.

At the same time, we would like to clarify that the d1T7-based sensing architecture implemented here differs mechanistically from previously established split-T7 systems. While both approaches ultimately rely on T7 RNAP-driven transcription, d1T7 employs a truncated promoter and recruitment of a full-length RNAP via DNA-binding domains, whereas split-T7 systems rely on reconstitution of an enzymatically active polymerase.

In this work, we extend the d1T7 framework beyond its original use in bacterial gene regulation by adapting it to a cell-free environment, expanding its regulatory scope, and implementing it as a biosensing platform for molecular diagnostics. We agree with the reviewer that these advances are best framed as system-level engineering and application development, and we have adjusted the manuscript accordingly.

We are grateful for the reviewer's positive assessment and helpful guidance in improving the clarity and positioning of our work.

Reviewer #3 (Remarks to the Author): Dear Authors,

Thank you for your detailed and thoughtful responses to our comments, as well as for the

substantial revisions made to the manuscript. We appreciate the care with which you addressed both the conceptual and technical concerns.

We find that the revised manuscript is significantly improved. In particular: - The authors have added new experimental data to support claims regarding multiplexing, matrix stability, and quantitative comparisons to wt T7RNAP. - Methodological clarity has been significantly improved, including clearer reporting of concentrations, additional details on PURE system preparation, and expanded explanations in the methods section. - Several conceptual criticisms regarding novelty, sensitivity, and applicability have been addressed through additional data and expanded discussion. Most significantly, the authors have clarified how their approach differs from prior split T7 approaches and outlined key advancements from the published d1T7 system. - Figures have been revised for clarity, and terminology (e.g. LOD vs. MDC) has been corrected where appropriate.

While some limitations remain (e.g. lack of clarity if Venetoclax could be monitored at home using minimally invasive blood collection and reliance on recombinant spike rather than virions), the revisions substantially improve transparency and technical rigor compared to the original submission.

Major concern: The authors failed to adequately address Reviewer 1's concern, regarding "The paper presents de novo design of functional protein binders (NB23 D1 and NB15 C1) using RFdiffusion, but there is no experimental binding data". Specificity is a critical issue in antibody engineering, particularly for weak binders, and without proper ELISA data it is difficult to conclude that these nanobodies are truly specific for the spike protein. Nanobodies should be tested against both the spike protein and a non-specific control antigen, alongside a non-specific antibody or nanobody as a negative control. The author's claim that "Our experiments show that signal is only generated in response to the addition of spike protein, our binders therefore do recognize and bind to spike." is not sufficient.

To assess specificity of our system, we conducted additional experiments challenging our de novo designed binder based sensing system with RSV F0 protein, a viral surface fusion protein from a distinct respiratory pathogen — as a non-cognate antigen control. As shown in Figure S8, the signal from RSV F0 (50nM) was statistically indistinguishable from the PBS negative control, while SARS-CoV-2 Spike (50nM) produced a significantly elevated signal relative to both RSV F0 and PBS. This pattern directly demonstrates that signal generation is driven by specific recognition of the Spike antigen rather than non-specific protein-induced background.

To further challenge binder specificity under conditions with higher complexity, we repeated the experiment in the presence of 10% nasal sample. Even under this condition, the system retained clear discrimination: Spike signal remained significantly elevated above both RSV F0 and PBS, while RSV F0 and PBS signals remained statistically equivalent (panels c–d). This demonstrates that binder selectivity is maintained in a complex matrix relevant to real diagnostic applications.

We also note that our binder selection was performed directly in the PURE cell-free system which contains highly concentrated ribosomes, proteins, and other biomolecules. The significant signal increase observed from the selected binders during the functional screen provides an additional, independent line of evidence that the selected binders engage their target through specific molecular recognition rather than non-specific adsorption.

Taken together, the RSV F0 cross-reactivity data — demonstrating no significant signal above PBS negative control in both buffer and nasal matrix conditions — alongside the stringent molecular background of the PURE-based binder selection, provide strong evidence that our de novo designed binders specifically recognize SARS-CoV-2 Spike protein.

At this stage, if the authors can provide experimental evidence to support the specificity of their computationally generated nanobodies, we will recommend publication in Nature Communications.

Reviewer #4 (Remarks to the Author): I co-reviewed this manuscript with one of the reviewers who provided the listed reports. This is part of the Nature Communications initiative to facilitate training in peer review and to provide appropriate recognition for Early Career Researchers who co-review manuscripts.
